# Intercurrent infection as a risk factor for disease flares in patients with systemic lupus erythematosus

**DOI:** 10.1136/lupus-2023-001131

**Published:** 2024-07-01

**Authors:** Fatma el Hadiyen, Michel W P Tsang-A-Sjoe, Birgit I Lissenberg-Witte, Alexandre E Voskuyl, Irene E M Bultink

**Affiliations:** 1 Department of Rheumatology, Amsterdam Rheumatology and immunology Center, Amsterdam UMC, Amsterdam, Noord-Holland, the Netherlands; 2 Department of Epidemiology and Data Science, VU University Amsterdam, Amsterdam, the Netherlands

**Keywords:** Systemic Lupus Erythematosus, Infections, Risk Factors, Disease Activity

## Abstract

**Objective:**

To determine whether intercurrent infections are a risk factor for subsequent disease flares in systemic lupus erythematosus (SLE).

**Methods:**

Demographic and clinical characteristics of 203 patients with SLE participating in the Amsterdam SLE cohort were collected at baseline and during follow-up. Collection of data on infections and SLE flares was registry-based and infections and flares were categorised as minor or major, based on predefined criteria. Proportional hazard models with recurrent events and time-varying covariates were used to estimate the HR of SLE flares.

**Results:**

The incidence rates of major and minor infections were 5.3 per 100 patient years and 63.9 per 100 patient years, respectively. The incidence rates of flares were 3.6 and 15.1 per 100 patient years for major flares and minor flares, respectively.

In the proportional hazard model, intercurrent infections (major and minor combined) were associated with the occurrence of SLE flares (major and minor combined; HR 1.9, 95% CI: 1.3 to 2.9). The hazard ratio for a major SLE flare following a major infection was 7.4 (95% CI: 2.2 to 24.6). Major infections were not associated with the occurrence of minor flares.

**Conclusions:**

The results of the present study show that intercurrent infections are associated with subsequent SLE flares, which supports the hypothesis that infections may trigger SLE flares.

**WHAT IS ALREADY KNOWN ON THIS TOPIC**

**WHAT THIS STUDY ADDS**

- Intercurrent infections are associated with subsequent SLE flares, which supports the hypothesis that infections may trigger SLE flares.

**HOW THIS STUDY MIGHT AFFECT RESEARCH, PRACTICE OR POLICY**

- This study promotes awareness of flares after infections and encourages further research into the underlying mechanisms.

## Introduction

 Intercurrent infections are a frequent and important comorbidity for patients with systemic lupus erythematosus (SLE), a chronic multisystem autoimmune disorder.^1^ The interplay between SLE and infections is complex. Aetiologically, several pathogens, especially Epstein-Barr Virus (EBV), Cytomegalovirus (CMV), parvovirus B19, human endogenous retroviruses, and to a lesser extent HIV and SARS-CoV-2 have been implicated in SLE pathophysiology.^2 3^ Clinically, symptoms accompanying intercurrent infections can mimic symptoms of SLE disease activity. Furthermore, patients with SLE are more susceptible to infections, probably due to the immunological perturbations leading to SLE, but certainly also due to treatment with immunosuppressants.

Previous observational studies suggested an increased rate of infections in patients with SLE compared with healthy controls.^4 5^ In a large Canadian population-based study, a twofold increased risk of severe infections was observed for patients with SLE compared with age-matched, gender-matched and index year-matched controls from the general population.^4^ In this study, severe infections were defined as infections that warranted hospital admission or infections that occurred during hospitalisation.^4^ In the most important prospective study to date, the relative risk of infection was found to be 1.63 times higher in the SLE group compared with the non-SLE group.^5^ The most common infection sites included the respiratory tract, urinary tract, skin and soft tissue and the bloodstream.^6^ The most commonly isolated pathogens responsible for these infections included *Staphylococcus aureus*, *Escherichia coli*, *Streptococcus pneumoniae*, *Varicella zoster*, *Cytomegalovirus and Candida*.^7^

The most frequently reported predictors of infection in patients with SLE were the use of glucocorticoids, the use of immunosuppressants and disease activity, especially in the case of lupus nephritis, serositis and haematological involvement.^6^

A widely accepted hypothesis is that intercurrent infections can provoke SLE disease flares.^8^ It has been proposed that intercurrent infections activate the immune system through pathways that are also involved in SLE pathophysiology, such as a type I interferon response, expelling neutrophil extracellular traps, increased rates of apoptosis and T-cell activation.^2 9 10^

However, data to support this hypothesis are surprisingly scarce. Clinical observational studies have identified bacteraemia, infection with influenza and varicella zoster infection as independent risk factors for disease flares.^11^,^14^ These studies were limited by their retrospective study design and only focused on a specific pathogen.

One of the few prospective cohort studies on infections and SLE flares demonstrated that 29% of patients experienced a worsening of their SLE following an infection.^5^ In this 3-year prospective study among 110 patients with SLE and 220 controls, infections were defined as clinical features supportive of an infection, positive blood cultures and/or response to antibiotic therapy.

In the current study, we aimed to examine, prospectively, the association between intercurrent infections and subsequent SLE disease flares.

## Methods

### Research design

Data from the Amsterdam SLE cohort were used for this study. The Amsterdam SLE cohort is an ongoing prospective, observational cohort study on disease course and outcome in SLE, which was initiated at VU University Medical Center (currently Amsterdam UMC), in 2007. Patients included in this study were followed from 2007 until 2016.

### Patient and public involvement statement

Two patients with experienced SLE research partners have been involved in the Amsterdam SLE cohort study since 2007.

### Study population

Patients with a diagnosis of SLE, who meet four or more criteria of the updated 1997 American College of Rheumatology (ACR) criteria^15^, and are at least 18 years of age were eligible for inclusion in the study cohort. After inclusion, patients have a yearly follow-up visit, during which data is collected on demographic and clinical characteristics, comorbidities, disease flares, treatment variables and intercurrent infections. During study visits, data is obtained by questionnaires, semi-standardised interviews, physical examination and laboratory tests. Patients were included in this substudy if they had had at least one follow-up visit. Written informed consent was obtained from all patients before their baseline visit.

### Data collection

At baseline and follow-up, the following data were collected: demographic and clinical characteristics, treatment-related variables and clinically relevant laboratory tests in blood and urine.

Demographic variables that were collected were: sex, ethnicity (Caucasian/not Caucasian), age at SLE onset and age at inclusion.

Clinical variables collected were: the number of ACR criteria at baseline^15^, body mass index, current alcohol and tobacco use and comorbidities (history of biopsy-proven lupus nephritis, renal insufficiency (defined as estimated glomerular filtration rate (eGFR)<45 mL/min), diabetes, (past) malignancies (with the exception of basal-cell carcinoma and squamous cell carcinoma), past cerebrovascular accident and asplenia (defined as past splenectomy or functional asplenia due to spleen infarctions). Disease activity was evaluated using the 2000 modification of the Systemic Lupus Erythematosus Disease Activity Index.^16^ Organ damage was assessed using the Systemic Lupus International Collaborating Clinics/ACR Damage Index (range 0–47).^17^ These scores were recorded during the yearly follow-up visits of the patients, in the same year as the year in which an intercurrent infection occurred.

Laboratory investigations consisted of complement C3/C4 levels, anti-double stranded DNA (anti-dsDNA) titre, total leucocyte count, lymphocyte count, neutrophil count, erythrocyte sedimentation rate, eGFR, serum 25(OH) vitamin D and fasting glucose level. Where possible, the results of laboratory investigations performed on the same day as the index date of the infection were used. If this was not possible, the most recent laboratory results before the index date of the infection were used for analysis.

Treatment variables that were assessed included glucocorticoid use (current use and mean daily dosage in milligrams), current use of hydroxychloroquine or other antimalarials, current use of azathioprine, mycophenolate mofetil, methotrexate, leflunomide, current cyclophosphamide use (used for at least 4 weeks at the index date of infection), current use of rituximab or belimumab (used for at least 3 months at the index date of infection) and current use of non-steroidal anti-inflammatory drugs (NSAIDs). These variables were collected at the date of hospital admission for a major infection. Because minor infections were treated in an outpatient setting, data on medication use at the time of a minor infection could not be retrieved for minor infections.

SLE flares were defined according to the definition proposed by Bootsma *et al*.^18^ Similarly to other SLE flare criteria, these criteria require an increase in disease activity combined with an intensification of immunosuppressive therapy. Flares were subsequently categorised into minor and major flares, depending on severity and required therapy. Frequency of flares, the severity of flares (minor/major), involved organ systems (mucocutaneous, musculoskeletal, haematological, neuropsychiatric, ophthalmological, cardiac, pulmonary, nephrological, constitutional or multiple organ systems), the time between index date of the infection and occurrence of flare (defined as the day on which immunosuppressive therapy was intensified) and intensification of treatment (addition of NSAID, addition of hydroxychloroquine or other antimalarials, addition of or increase in the dose of a glucocorticoid, addition of or switch to another disease-modifying anti-rheumatic drug (DMARD) or addition of a biological DMARD to the maintenance therapy of the patient) were collected. Data on SLE flares were collected during follow-up.

Major infections were defined as infections for which hospital admission and/or intravenous antibiotic therapy was required. Minor infections were defined as infections for which hospital admission was not warranted, but were regarded or treated as such by a treating physician.^19^ Infections were confirmed by clinical findings and/or a positive culture and/or response to therapy. The risk interval for the occurrence of a disease flare was defined as 3 months from the index date of an infection. This interval was chosen from a clinical point of view and was in line with a few earlier studies on the same topic.^11 12^ The following data were collected retrospectively by chart review: the index date of infection (defined as the day on which treatment was started and/or the day on which the patient was admitted to hospital), severity of infection (minor/major), the involved organ system(s) (skin and soft tissue, musculoskeletal, central nervous system, otorhinolaryngologic, ophthalmologic, respiratory tract, cardiac, gastrointestinal, intra-abdominal, urinary tract, genital tract or bloodstream), the involved pathogens (viruses, bacteria, fungi, yeasts or parasites), mortality causes (involved pathogen(s) and organ system(s)), treatment (classification of medication and specific agent), treatment duration and admission to hospital (yes/no) in the previous year since the last follow-up appointment.

### Statistical analysis

The incidence rate of disease flares following a major or minor infection, and their corresponding 95% CI were assessed. In addition, the incidence rates and corresponding 95% CIs of major and minor infections, as well as the incidence rates of major and minor flares that occurred during follow-up were calculated. Incidence rates were presented as a number of events per 100 patient-years. Descriptive analyses were applied where appropriate. Proportional hazard models with recurrent events and time-varying covariates were used to estimate the HRs of SLE flares to determine whether intercurrent infection acts as a risk factor for SLE flare. The value of time-varying covariate infection was set to 1 for the time between the start of the infection and 3 months later, whereas the value was set to 0 for the time 3 months after the infection until the next infection (or end of follow-up). The models were corrected for confounding variables if the confounding variable (1) was associated with both intercurrent infection as well as SLE flares, and (2) changed the regression coefficient of intercurrent infection by more than 10%. Cumulative incidence curves for the incidence of flares were estimated using the proportional hazard models. The curve for ‘infection’ corresponds to a hypothetical patient who has an infection from cohort entry until the end of follow-up, while the curve for ‘no infection’ corresponds to a hypothetical patient, who does not have an infection during the follow-up period. SPSS V.26 was used for descriptive analysis of the data (SPSS, Chicago, Illinois, USA). R V.4.0.3 was used for longitudinal analysis of the data.

## Results

At the time of analysis, sufficient data was available for 203 patients in the cohort. Included patients had a median (IQR) follow-up duration of 6 years and a total follow-up of 1060 patient-years. Table 1 shows the demographic and clinical characteristics of the patients. Most of the patients were Caucasian and women. Disease activity at cohort entry was generally low. The majority of the patients were treated with antimalarials. Half of the patients were treated with glucocorticoids of which 64% (68/106) were using a prednisolone dose above 7.5 mg/day.

**Table 1:** Baseline demographic and clinical characteristics

Variables	Patients with SLE (n=203)
Demographic variables	
Sex, female, n (%)	184 (91)
Age, years (median (IQR))	40.0 (32.0–47.0)
Caucasian ethnicity, n (%)	137 (68)
Body mass index, kg/m^2^ (median (IQR))	23.3 (21.2–26.9)
Current smoker, n (%)	38 (19)
Current alcohol user, n (%)	97 (48)
Clinical variables	
SLICC/ACR Damage Index (median (IQR))	1 (0–2)
SLEDAI2k (median (IQR))	4 (2–6)
Disease duration, years (median (IQR))	6 (1–11)
History of:	
Biopsy-proven lupus nephritis, n (%)	38 (19)
Renal insufficiency, n (%)	6 (3)
Diabetes mellitus, n (%)	7 (3)
Malignancy, n (%)	5 (3)
Stroke, n (%)	12 (6)
Asplenia, n (%)	5 (3)
Treatment variables:	
Glucocorticoids, n (%)	106 (52)
Antimalarials, n (%)	151 (74)
Azathioprine, n (%)	51 (25)
Cyclophosphamide (oral), n (%)	1 (1)
Cyclophosphamide (IV), n (%)	3 (2)
Methotrexate, n (%)	8 (4)
Mycophenolate mofetil, n (%)	11 (5)
Rituximab, n (%)	1 (1)
NSAIDs, n (%)	67 (33)

IV, intravenous; NSAID, non-steroidal anti-inflammatory drugsSLEsystemic lupus erythematosusSLEDAI2k, Systemic Lupus Erythematosus Disease Activity Index 2000; SLICC/ACR, Systemic Lupus International Collaborating Clinics/American College of Rheumatology

### Major infections

A major infection occurred in 30 out of 203 patients during follow-up. Multiple severe infections in the same patient were common. 12 patients had more than one severe infection, 4 of whom had more than three severe infections. In total, 56 major infections occurred in 1060 patient-years, which yielded an incidence rate of 5.3 per 100 patient-years (95% CI: 4.1 to 6.9). 23% (7/30) of patients who had a major infection during follow-up had a history of biopsy-proven lupus nephritis at baseline.

In table 2, the organ system distribution of major infections is shown. Most of the major infections involved the respiratory tract, followed by the urinary tract, the bloodstream and skin and soft tissue. Within the group of the respiratory tract infections, one of the major infections was classified as a pneumosepsis with a blood culture that was positive for *S. aureus*. Out of 14 major infections in the urinary tract group, there was 1 case of urosepsis with a positive blood culture that showed *E. coli*.

**Table 2:** Localisations of major and minor infections

Localisation of infection	Major infections (n=56)	Major infections followed by flares within 3 months (n=7)	Minor infections (n=670)	Minor infections followed by flares within 3 months (n=24)
Respiratory tract	13	0	203	7
Urinary tract	14	2	146	8
Soft tissue	5	0	99	3
Oropharyngeal	5	2	81	2
Gastrointestinal	5	2	29	0
Eye	1	0	24	0
Genital	1	0	55	1
Dental	0	0	19	2
Intra-abdominal	3	0	4	0
Musculoskeletal	1	0	2	0
Systemic	2	0	3	1
Bloodstream	6	1	0	0
Cardiac	0	0	0	0
Central nervous system	0	0	0	0
Not specified	0	0	5	0

Most of the major infections were caused by bacteria. The most frequently identified micro-organism was *E. coli*, followed by *S. aureus*, *Coagulase negative staphylococci* and *S. pneumoniae.* Other bacteria that were identified include: *Chlamydia trachomatis*, *Staphylococcus epidermidis*, *Bacteroides, Stenotrophomonas maltophilia and Haemophilus influenza*, *Citrobacter koserii*, *Beta hemolytic streptococcus group A*, *Clostridium difficile*, *Gardnerella vaginalis*, *Staphylococcus capitis*, *Serratia marcescens*, *Salmonella group C*, *Aeromonas hydrophila* and *Pseudomonas aeruginosa.* In two cases, viruses were the cause of the major infection. In one case, *herpes simplex* was identified, while in the other case *parainfluenza* 3 was isolated. In 39% of the major infections, no pathogen was identified.

Table 3 shows medication use at the index date of a major infection. In six cases, patients were not using SLE medication. For six other major infections, information about medication use at the index date of the infection could not be retrieved. In most cases, patients were using glucocorticoids, while antimalarials were used in less than half of the cases. In 61% of the cases, patients were using DMARDs.

**Table 3:** Medication use at the index date of a major infection in 30 patients with systemic lupus erythematosus

Medication agent	Use at onset of major infection n=56 (n, %)
Glucocorticoids	38 (68)
Antimalarials	26 (46)
Azathioprine	13 (23)
Mycophenolate mofetil	14 (25)
Methotrexate	7 (13)
NSAIDs	3 (5)
No medication	6 (11)
Unknown	6 (11)

NSAIDs, non-steroidal anti-inflammatory drugs

In 38 cases, there was no change in medication use during the course of the infection. In 11 cases, the glucocorticoid dose was increased during the course of the major infection. DMARDs were temporarily stopped in six cases. In one case, a DMARD was switched to another.

### Minor infections

In total, 670 minor infections occurred in 157 out of 203 patients in 1048 patient years during follow-up, which yielded an incidence rate of 63.9 per 100 patient years (95% CI: 59.3 to 69.0). Data could not be retrieved for two patients who were subsequently excluded from the analysis. 23% (36/157) of patients who had a minor infection during follow-up, had a history of biopsy-proven lupus nephritis at baseline.

The distribution of affected organ systems is shown in table 2. Like major infections, most of the minor infections occurred in the respiratory and urinary tracts. Other frequent localisations for minor infections include the skin and soft tissue, the eye, the oropharyngeal tract, the dental, gastrointestinal tract and the genital organs.

For minor infections, yeasts were the most commonly isolated pathogens, followed by viruses and bacteria. *Candida albicans* was responsible for all minor yeast infections that were cultured. Most viral minor infections (28 out of 44) were caused by *V. zoster,* while *norovirus*, *herpes simplex* and *human papillomavirus* accounted for four viral minor infections each. Other viruses that were isolated are *dengue*, *CMV, parvovirus B19* and *hepatitis C.* 9 out of 28 bacterial minor infections were caused by *E. coli,* while *Klebsiella pneumoniae* was responsible for four infections. Other bacteria that were found were *Salmonella, S. aureus, H. influenza, Proteus mirabilis, Staphylococcus saprophyticus, G. vaginalis, S. pneumonia, P. aeruginosa, Enterobacter, P. mirabilis, Borrelia burgdorferi.* The involved pathogen remained unknown in 81% of minor infections.

### Flares

In total, 198 flares occurred during 1060 patient years, 38 of which were classified as major flares and 160 as minor flares. The overall incidence rate of flares during follow-up was 18.7 per 100 patient-years (95% CI: 16.3 to 21.5). The incidence rate of major flares and minor flares was 3.6 and 15.1 per 100 patient-years (95% CI: 2.6 to 4.9 and 95% CI: 12.9 to 17.6), respectively. In the case of major flares, 19 patients had one flare, 5 patients had two flares and 3 patients had three flares. In the case of minor flares, 33 patients had one flare, 25 patients had two flares and 17 patients had three or more flares. The distribution of flares among the organ systems is shown in online supplemental table 1. The most frequently involved organ system in major flares was the kidney, accounting for 42% of all major flares. The musculoskeletal and mucocutaneous organ systems were the most frequently involved organ systems in minor flares (41% and 24%, respectively). Multiple organ involvement was present in 14% of minor flares.

### Intercurrent infections followed by an SLE flare within 3 months

7 out of 56 major infections (12.5%) were followed by an SLE flare within 3 months. One patient had two major infections that were followed by a flare within 3 months. In only one out of seven of these infections the immunosuppressants were temporarily withdrawn. In three cases, the glucocorticoid dose was increased, and in three other cases no changes were made in SLE medication use during the course of the infection.

In 3.5% of minor infections (24/670) an SLE flare occurred within 3 months. Two patients had four minor infections that were followed by a flare and one patient had two minor infections that were followed by a flare.

Online supplemental table 2 demonstrates the pathogens that were involved in infections that were followed by a flare within 3 months.

**Figure 1:**
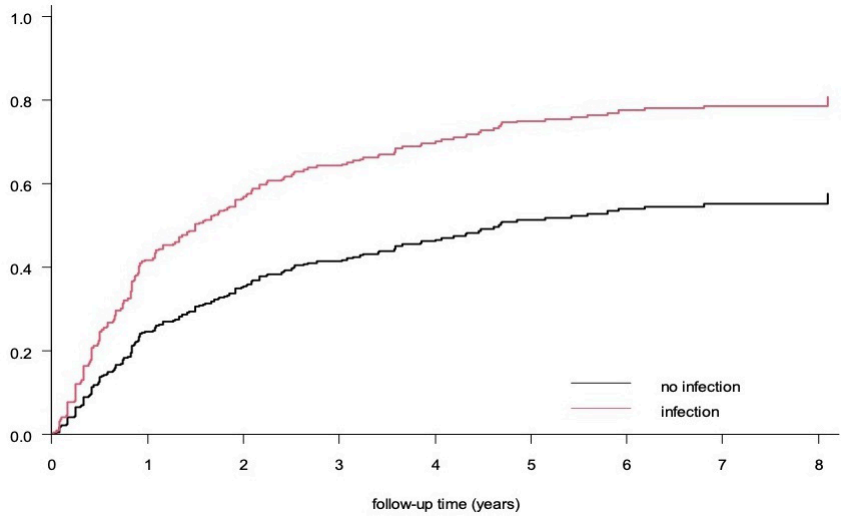
These curves are estimated from the survival model. The curve for ‘infection’ corresponds to a hypothetical patient, who has an infection from cohort entry until the end of follow-up, while the curve for ‘no infection’ corresponds to a hypothetical patient, who does not have an infection during the follow-up period.

**Figure 2:**
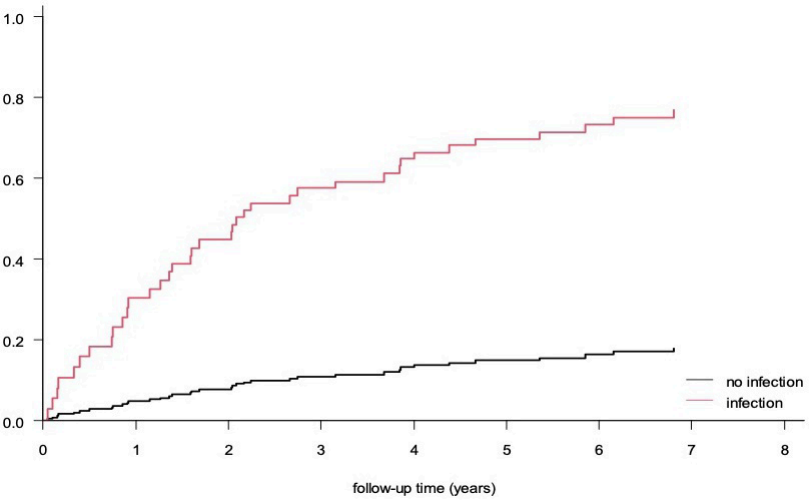
These curves are estimated from the survival model. The curve for ‘infection’ corresponds to a hypothetical patient, who has an infection from cohort entry until the end of follow-up, while the curve for ‘no infection’ corresponds to a hypothetical patient, who does not have an infection during the follow-up period.

To assess whether there is an increased risk of SLE flares after intercurrent infections, we constructed a proportional hazards model. In this model, intercurrent infections (both major and minor) were indeed associated with SLE flares (both major and minor) within 3 months after the index date of the infection (HR=1.9; 95% CI: 1.3 to 2.9) (figure 1). Correction for any of the demographic variables in table 1 was not needed. In subsequent analyses, the association with the highest risk was found for the occurrence of major flares after a major infection (HR=7.4; 95% CI: 2.2 to 24.6) (figure 2). We also observed a statistically significant association between intercurrent infections (both major and minor) and minor SLE flares (HR=1.9; 95% CI: 1.2 to 3.0). However, no such association was found between intercurrent infections (both major and minor) and major flares (HR=2.1; 95% CI: 0.9 to 5.1).

## Discussion

In this prospective cohort study an HR of 1.9 was found for SLE flares (major and minor combined) occurring within 3 months after an intercurrent infection (major and minor). The highest risk (HR=7.4) was found for major flares within 3 months after a major infection. These findings support the hypothesis that intercurrent infections are a potential trigger for disease flares and is in line with other observational studies that focused on a single pathogen.^11^,^14^

In our study, the incidence rates of major and minor infections were 5.3 and 63.9 per 100 patient-years, respectively. In a Canadian prospective cohort study, an incidence rate of 3.8 per 100 patient-years was found for severe infections.^4^ The results of a large US-based cohort study in patients with SLE demonstrated an incidence rate of 10.8 per 100 patient years for major infections.^20^ The definition of severe infections used in those studies was the same as the definition of major infections used in our study. The differences in the incidence rates of infections between the studies might be related to several factors, such as differences between the endemicity of certain infections, differences in vaccination strategies between countries and differences in ethnic background, socioeconomic status, access to healthcare, disease severity and medication use between study populations. For example, in our study, 74% of patients were using antimalarials at baseline, while the US-based study reported 39% ever antimalarial use in their study population. Hydroxychloroquine has previously been reported to be protective against infections and is recommended in current guidelines for SLE management.^19^,^23^ Nonetheless, these high incidence rates of severe infections underline the importance of infections as a major comorbidity in SLE.

Moreover, the results of our study demonstrate that infections are an important risk factor for the occurrence of a subsequent SLE flare, while previous studies have demonstrated that disease flares are in turn associated with increased morbidity and mortality and a reduced quality of life in patients with SLE.^24^,^26^ In particular, major infections were associated with a 7.4 times increased risk of the occurrence of a major SLE flare within 3 months. This finding stresses the importance of awareness and strict monitoring of disease activity in patients with SLE suffering a major infection, and prompt adequate treatment in case of the development of a disease flare.

In the present study, no pattern could be discerned with regard to a specific pathogen or organ system distribution of the infections and the occurrence of certain SLE flares.

We hypothesised that discontinuation of immunosuppressive agents during an active major infection might, in part, explain the subsequent occurrence of SLE flares. However, the results of our study did not support this hypothesis, because temporary withdrawal of immunosuppressive agents during a major infection was executed in only one out of seven major infections which were followed by a disease flare within 3 months.

The present study has a number of strengths. It is a relatively large prospective study, using real-world data that includes an assessment of intercurrent infections and disease flares. In addition, while most studies on this topic focused on a specific pathogen, our study included all incident infections during the follow-up period. A limitation of our study is that data on minor infections are in part limited due to recall bias. In addition, the number of patients with infections in whom causative organisms were identified was small. In our study, we assessed minor infections through detailed yearly interviews with participants of the cohort. In the Netherlands most minor infections are diagnosed and treated by primary care physicians and most patients with SLE will consult their primary care physician first, before contacting their rheumatologist. Despite these limitations, the results of our study may provide an impression of the burden of minor infections in SLE. Data on minor infections in SLE are very scarce as healthcare insurance databases or retrospective analyses of hospital records are insufficient. Another limitation of our study is the fact that we could not account for the possible effects of certain antibiotic agents that are known to induce lupus flares or drug-induced lupus in our model. Unfortunately, our study population was too small to investigate associations between the use of specific types of antibiotic agents and the risk of disease flares in our cohort. However, only 1 of the 56 major infections in our study population was treated with an antibiotic agent that has been associated with drug-induced lupus (nitrofurantoin) and this infectious episode was not followed by a disease flare within 3 months. Furthermore, differences in immunosuppressive drugs used at the time of infection and temporarily withdrawal of these drugs during an infectious episode might have influenced the risk of disease flares in the next 3 months. In our study, in only one out of seven major infections followed by a disease flare, immunosuppressive agents were withheld at the start of the infection. Therefore, the number of events was too small in our study to account for this factor in the model. Another limitation is that the Bootsma criteria were used to define SLE flares. These criteria are not commonly used in more recently initiated international cohort studies. At the time of the start of this cohort study in 2007, there was no real consensus on which definition of SLE flares to use, so, we ultimately decided to use the Bootsma criteria which were designed according to the same principles as other more recently constructed criteria to define SLE flares. Finally, the association between the use of newer agents for the treatment of SLE, such as rituximab or belimumab and the occurrence of infections and subsequent disease flares could not be studied separately, as these agents were rarely prescribed in our cohort during the study period (2007 until 2016).

In conclusion, intercurrent infections, both major and minor, are frequent in SLE. Our finding, that patients with SLE who suffer an intercurrent infection are at increased risk of developing an SLE flare in the following 3 months warrants closer monitoring.

## supplementary material

10.1136/lupus-2023-001131 online supplemental table 1

10.1136/lupus-2023-001131 online supplemental table 2

## Data Availability

Data are available upon reasonable request.
