# Supplementary material for: Intercurrent infection as a risk factor for disease flares in patients with systemic lupus erythematosus
Source: Lupus Sci Med. 2024 Jul 1;11(2):e001131. doi: 10.1136/lupus-2023-001131 (PMC11217993; doi:10.1136/lupus-2023-001131)
Supplement: online supplemental table 1 [file lupus-2023-001131-s001.pdf]

Table S1 Organ systems involved in major flares (n=38) and minor flares (n=160) in 86 SLE patients

**Organ system**	**Number of major flares (n, %)**	**Number of minor flares (n, %)**
Nephrological	16 (42)	0 (0)
Neuropsychiatric	6 (16)	0 (0)
Pulmonary	4 (11)	12 (8)
Cardiac	3 (8)	11 (7)
Mucocutaneous	2 (5)	39 (24)
Musculoskeletal	2 (5)	66 (41)
Hematological	2 (5)	4 (3)
Ophtalmological	1 (3)	1 (1)
Constitutional	0 (0)	4 (3)
Miscellaneous	0 (0)	1 (1)
Multiple organ systems	2 (5)	22 (14)
