# Supplementary material for: Intercurrent infection as a risk factor for disease flares in patients with systemic lupus erythematosus
Source: Lupus Sci Med. 2024 Jul 1;11(2):e001131. doi: 10.1136/lupus-2023-001131 (PMC11217993; doi:10.1136/lupus-2023-001131)
Supplement: online supplemental table 2 [file lupus-2023-001131-s002.pdf]

Table S2 Pathogens involved in infections that were followed by a flare within three months

**Major infections (n=7)**		**Minor infections (n=24)**	
**Pathogen**	**No. of episodes**	**Pathogen**	**No. of episodes**
Bacteria<br>- *E. coli*<br>- *Bacteroides*<br>- *Mixed flora*	3<br>1<br>1<br>1	Bacteria<br>- *E. coli*<br>- *Pseudomonas aeruginosa*<br>- *Salmonella*	6<br>3<br>2<br>1
Viruses<br>- *Herpes simplex*	1<br>1	Viruses<br>- *Human papilloma virus*	2<br>2
Yeasts	0	Yeasts<br>- *Candida albicans*	1<br>1
Unknown	3	Unknown	15
